# Legacy habitat contamination as a limiting factor for Chinook salmon recovery in the Willamette Basin, Oregon, USA

**DOI:** 10.1371/journal.pone.0214399

**Published:** 2019-03-22

**Authors:** Jessica I. Lundin, Julann A. Spromberg, Jeffrey C. Jorgensen, James M. Myers, Paul M. Chittaro, Richard W. Zabel, Lyndal L. Johnson, Robert M. Neely, Nathaniel L. Scholz

**Affiliations:** 1 National Research Council Research Associateship Program, Under contract to Northwest Fisheries Science Center, National Marine Fisheries Service, National Oceanic and Atmospheric Administration, Seattle, WA, United States of America; 2 Environmental and Fisheries Science Division, Northwest Fisheries Science Center, National Marine Fisheries Service, National Oceanic and Atmospheric Administration, Seattle, WA, United States of America; 3 Fish Ecology Division, Northwest Fisheries Science Center, National Marine Fisheries Service, National Oceanic and Atmospheric Administration, Seattle, WA, United States of America; 4 Conservation Biology Division, Northwest Fisheries Science Center, National Marine Fisheries Service, National Oceanic and Atmospheric Administration, Seattle, WA, United States of America; 5 Assessment and Restoration Division, Office of Response and Restoration, National Ocean Service, National Oceanic and Atmospheric Administration, Seattle, WA, United States of America; Fisheries and Oceans Canada, CANADA

## Abstract

In the western United States, the long-term recovery of many Pacific salmon populations is inextricably linked to freshwater habitat quality. Industrial activities from the past century have left a legacy of pollutants that persist, particularly near working waterfronts. The adverse impacts of these contaminants on salmon health have been studied for decades, but the population-scale consequences of chemical exposure for salmonids are still poorly understood. We estimated acute and delayed mortality rates for seaward migrating juvenile Chinook salmon that feed and grow in a Superfund-designated area in the Lower Willamette River in Portland, Oregon. We combined previous, field-collected exposure data for juvenile Chinook salmon together with reduced growth and disease resistance data from earlier field and laboratory studies. Estimates of mortality were then incorporated into a life cycle model to explore chemical habitat-related fish loss. We found that 54% improved juvenile survival—potentially as a result of future remediation activities—could increase adult Chinook salmon population abundance by more than 20%. This study provides a framework for evaluating pollution remediation as a positive driver for species recovery.

## Introduction

Pacific salmon (*Oncorhynchus* spp.) habitat degradation and loss in the large river basins of the Pacific Northwest began with settlements soon after the formation of the Oregon Territory in 1848 and steadily continued for the ensuing 150 years [1]. In addition to blocking migratory access (e.g., dams, dikes, culverts), human activities have negatively altered numerous physical and biological habitat features, including flow regimes, surface water temperatures, and floodplain connectivity [2]. Based on historical estimates, 29% of Pacific salmon species (distinct population segments) have been extirpated due to these and other habitat-related drivers [3]. Of the salmon populations that remain, more than two-thirds are presently listed as either threatened or endangered under the U.S. Endangered Species Act (ESA) [4].

More broadly, efforts to reverse salmon declines have largely focused on regulated harvests (allowable catch), hydropower modifications, hatchery reform, and habitat restoration. In recent decades, billions of U.S. dollars have been spent on freshwater and estuarine habitat restoration projects [5, 6]. These improvements have focused almost exclusively on physical and biological processes. Examples include the removal of dams, culverts, and other migration barriers; the reestablishment of hydrologic, riparian, and geologic processes; and the addition of structural enhancements such as large woody debris or boulders [5, 7]. The desired outcomes are an expanded range, increased flows, cooler surface waters, increased access to sheltered areas, and increased prey availability for rearing and outmigrating juveniles, as well as returning adults. To date, however, these cumulative restoration actions have not been enough to boost population abundances to an extent that justifies ESA delisting. This suggests that other, non-physical habitat attributes may be limiting salmon recovery to a greater extent than previously appreciated.

Salmon are exposed to a range of potentially toxic chemical contaminants at different points during their highly migratory life histories [8]. Salmon habitats are widely degraded by chemical pollution from legacy industrial activities, modern agriculture, urban stormwater runoff, municipal wastewater discharges, and accidental spills [9]. Moreover, chemical pollution can undermine the success of physical restoration projects [10]. While rare, acute mortality events have been documented in salmon habitats (e.g., Scholz et al. 2011). Most contaminant exposures, by contrast, are sublethal and include such adverse effects such as compromised immune function, decreased growth, and impaired reproduction. These types of delayed or protracted impacts on salmon health may nevertheless reduce the lifetime survival and reproductive success of individual fish, thereby reducing population abundance [11]. The life histories of anadromous Pacific salmon species can be an important determinant of contaminant exposure [12]. For example, whereas chum (*O*. *keta*) and pink (*O*. *gorbuscha*) salmon, and ocean-type Chinook salmon (*O*. *tshawytscha*), emigrate to estuarine waters relatively soon after hatch, coho (*O*. *kisutch*) and stream-type Chinook salmon (*O*. *tshawytscha*) juveniles rear in natal freshwater streams for up to two years [13]. Therefore, pollution reduction efforts in freshwater habitats may have different impact on salmonids depending on their life-history strategies, with stream-type salmonids likely to benefit the most from pollution reduction efforts.

There have been limited attempts to model the impacts of suborganismal toxicity at the scale of wild salmon populations [14–16]. Also lacking are analytical frameworks for directly comparing the population-scale influences of chemical and physical habitat degradation, as a basis for side-by-side evaluations of limiting factors for salmon recovery as well as prioritizing future restoration activities. This would be most tractable for a data-rich salmon population, with sufficient life-history information such as survival probability at each life stage, demographic abundance data, and spatial and temporal distributions. Life-history information could then be combined with known habitat characteristics–physical, biological, and chemical–in a demographic model to evaluate the relative benefits of different types of habitat improvements. As previously described, this would involve the translation of sublethal toxicity and delayed mortality to the population scale [15, 17]. Demographic models are increasingly in use to support recovery decision making for ESA-listed salmonids [18, 19]. However, as noted earlier, the current models do not directly address degraded water or sediment quality as a result of toxic pollutants.

**Fig 1:**
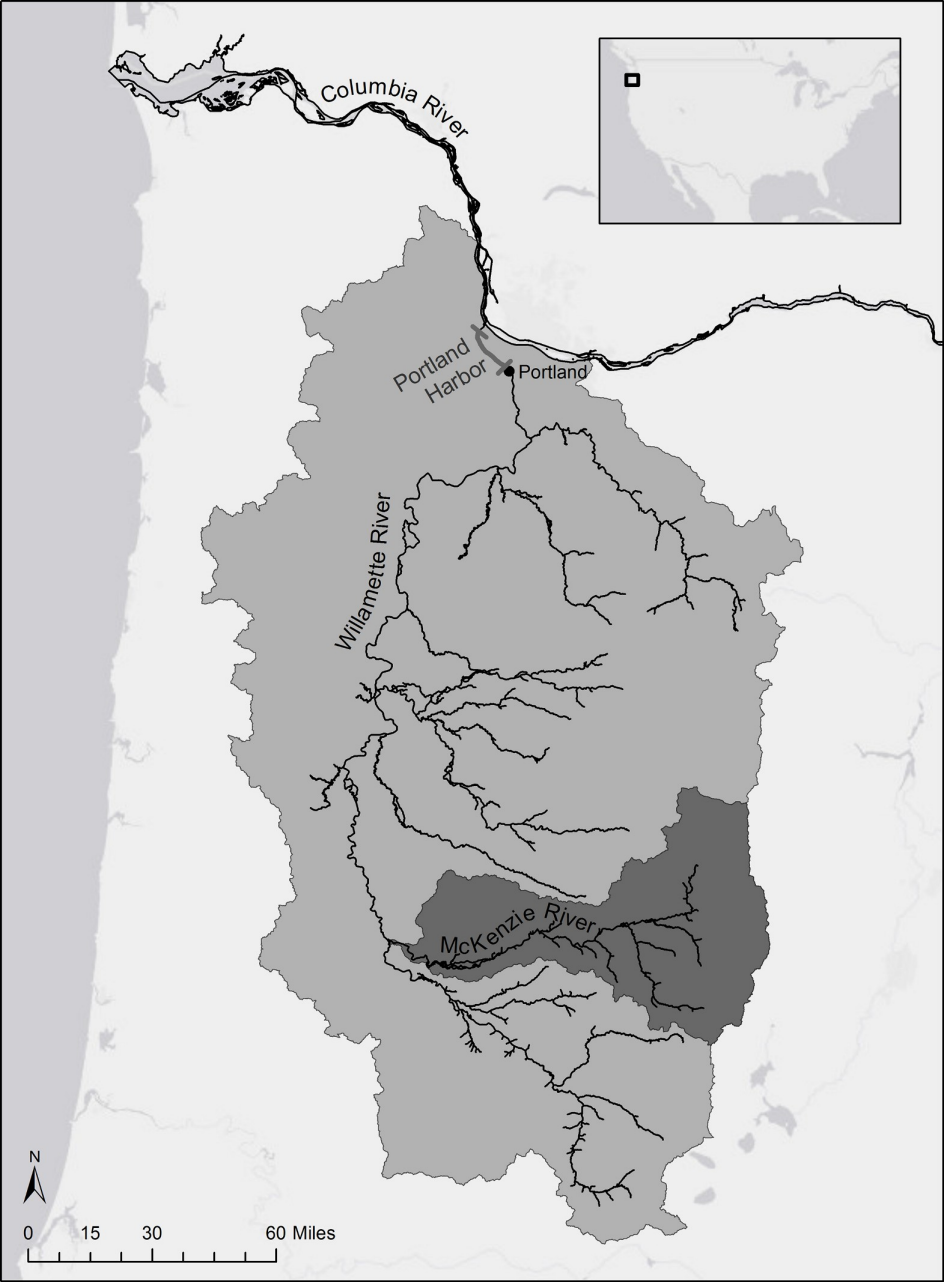
Map of McKenzie River watershed within Willamette River basin. Portland Harbor is bounded by grey dashes.

Here we model the impact of legacy chemicals on ESA-listed Chinook salmon (*O*. *tshawytscha*) that spawn in the upper reaches of the Willamette River Basin (henceforth referred to as Upper Willamette River; UWR) in Oregon, USA. Juveniles migrate seaward through historically contaminated Portland Harbor, a designated Superfund site since 2000 (described in detail below). Populations of Chinook salmon in the UWR are at historically low abundances, and were designated as “threatened” under the U.S. Endangered Species Act in 1999 [20]. The recovery of the Chinook salmon populations, collectively designated as the Upper Willamette Evolutionarily Significant Unit (ESU), has been a long-term priority for NOAA Fisheries and other regional stakeholders, and has been an impetus for in-depth analyses of freshwater habitats in terms of both physical attributes and Chinook salmon use patterns [18, 21, 22]. Upper Willamette Chinook salmon are comprised of seven populations. The focal salmon for this study were the McKenzie River Spring Chinook salmon population (Fig 1) of the UWR ESU. The McKenzie River population is the only self-sustaining naturally-spawning UWR ESU population, including about 26% hatchery fish [23]. Other populations have an estimated hatchery fraction of over 75%, with the exception of North Fork Clackamas River spring chinook with an estimated fraction of 64% [23]. Approximately 18% of emigrating McKenzie River fish (7 year average between 2004 and 2013) in the spring are the smaller young of the year juveniles (subyearlings), 13% of juveniles emigrate in the fall, and the remaining 69% emerge the following spring as yearlings [21]. Tagging studies show the yearlings spend an estimated 2–4 days in Portland Harbor as they outmigrate [22]. The subyearlings are too small to tag, but it has been estimated the UWR subyearlings spend weeks or longer in Portland Harbor to feed and grow before transitioning to the lower Columbia River estuary [22].

**Fig 2:**
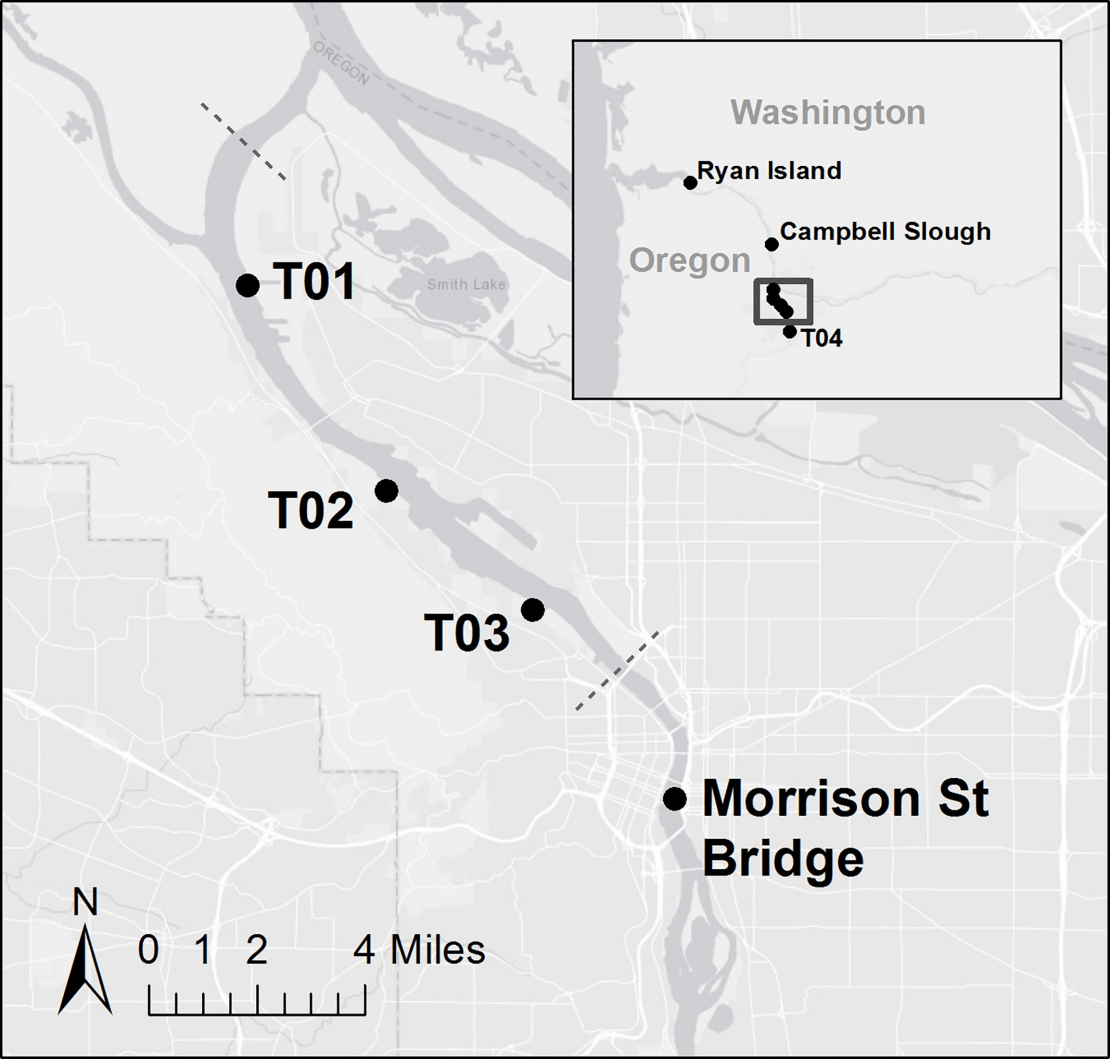
Map of juvenile Chinook salmon sampling sites within Portland Harbor. Inset shows up- and downstream sampling sites. Dashed lines show approximate boundaries of Superfund Site [26, 27].

The Willamette River flows north through the highly industrialized Portland Harbor prior to its confluence with the lower Columbia River (Fig 1). For more than a century, this harbor has functioned as a commercial shipping port and working waterfront. Over the decades, numerous industries have released toxic chemicals into the river. Common sources of pollution have included permitted and non-permitted end-of-pipe discharges, accidental spills during cargo transfers, and stormwater and groundwater transport from upland areas [24]. Extensive legacy pollution in harbor sediments eventually led the U.S. Environmental Protection Agency (EPA) to add Portland Harbor to the Comprehensive Environmental Response, Compensation, and Liability Act (CERCLA) National Priorities List (i.e., designated Superfund site) in December 2000. At present, the Superfund site extends from river mile 2 to 11 (dashed lines, Fig 2), inclusive of upland areas. Priority contaminants of concern include polychlorinated biphenyls (PCBs), organochlorine pesticides including dichloro-diphenyl-trichloroethanes (DDTs), polycyclic aromatic hydrocarbons (PAHs), and antifouling agents such as butyltins [e.g., tributyltin (TBT)]. In March 2017, the U.S. EPA issued the Record of Decision for clean-up of the site to include active remediation of contaminated sediment and river banks to reduce risks to human health and the environment, which will take an estimated 13 years to complete [25].

Our approach applied a Chinook salmon life cycle model in a novel way to evaluate population level impacts of legacy chemicals. The model was developed to address physical and biological habitat factors associated with proposed dam alternatives affecting this McKenzie River Spring Chinook salmon population [18]. We parameterized the population model to incorporate chemical habitat characteristics. The best available science was used to make simple assumptions on loss of subyearling outmigrants due to acute and delayed mortality as a consequence of tissue concentrations of contaminants associated with the relatively protracted residence time interval of feeding and growth, notably in close proximity to the riverbank edges [28], in industrially contaminated Portland Harbor. The remaining juvenile Chinook salmon (older and larger yearlings) outmigrants move directly to the estuary after spending only a few days in Portland Harbor [22]; therefore were not considered in this study. This approach allowed us to demonstrate the utility of this modeling approach to assess reductions in direct and sublethal toxicity to subyearling juveniles, on a population-scale.

## Methods

### Juvenile Chinook salmon collection and tissue residue values

Estimates of contaminant exposure in the form of tissue residues (body burdens) were obtained from previous field collections of juvenile Chinook salmon at multiple sites throughout, and downstream of, Portland Harbor. Briefly, in May 2005 the Lower Willamette Group (http://lwgportlandharbor.org) captured subyearlings by beach seine from three locations within the Superfund site (i.e., referred to as sample sites T01-T03), as well as an upstream site at river mile 17–18 (site T04) (Fig 2) [27]. Seining was selective for fish in the 50–80 mm fork length size range. Three replicate composite samples of 30 whole-fish (stomach contents removed) were collected, along with 1–2 composite samples of stomach contents per site.

In addition, as part of a previous longitudinal study, the Lower Columbia River Estuary Partnership (http://www.estuarypartnership.org) collected juvenile Chinook salmon in the lower Willamette and Columbia Rivers for chemical analyses. Monthly sampling from 2005–2009 yielded 1,200 juveniles, primarily of fork length < 100 mm, from thirty separate sampling events at 15 sites, for a total of 122 composite samples (3–10 individual fish per composite) [26]. Stock of origin for all fish was confirmed by conventional genetic analysis, as described by Teel et al. [29]; two sampling events below the Columbia-Willamette confluence yielded enough fish for whole body composite sample analysis of UWR Chinook (Campbell Slough, May 2007; Ryan Island, May 2009) (Fig 2). Stomach content samples were composited by site of collection, not genetic stock of juvenile Chinook. Stomach contents from juvenile Chinook collected from Campbell Slough were composited into three samples; no data on stomach contents were available from Ryan Island (S1 Table). The Morrison Street Bridge location, just upstream from Portland Harbor, was sampled in 2005 (April, May, and June) [26] and June 2013 (unpublished data; continuation of 2005–2009 study using same methods and protocols) [30] (Fig 2). These two collection efforts upstream of the Superfund site each produced four whole body composite samples of genetically confirmed Upper Willamette Chinook, and 8 site-based stomach content composite samples.

**Fig 3:**
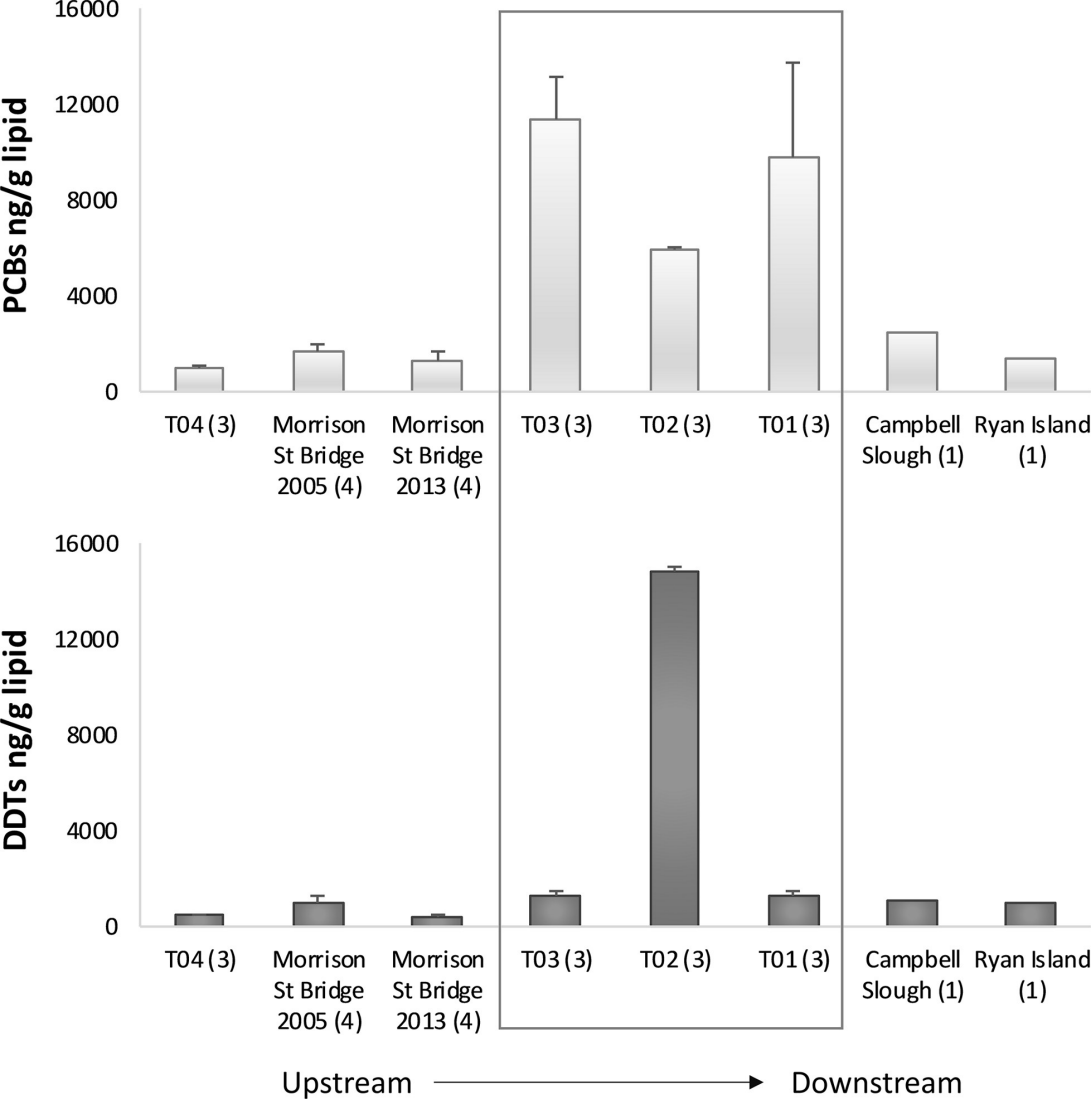
Measured persistent organic pollutants (PCBs and DDTs) in the tissues of outmigrating UWR juvenile Chinook salmon [26, 27, 30]. Mean values from whole body composite samples (n), with the stomach contents removed, from three sites within Portland Harbor (box), two sites upstream, and two sites downstream. Error bars show standard deviation. For consistency across studies, PCBs reported as ∑17PCBs*2 (PCBs 18, 28, 44, 52, 95, 101, 105, 118, 128, 138, 153, 170, 180, 187, 195, 206, 209); DDTs reported as ∑3DDTs (p,p’-DDD, p,p’-DDE, p,p’-DDT). Figure data and data for lipids, butyltins, and PAHs on S1 Table.

Samples from both studies were analyzed for PCBs, DDTs, PAHs, and percent lipids. Butyltins were only measured in the samples T01-T04, thus TBT exposure evaluations were limited to these samples. The analytic methods of chemical analysis for both studies is available in the supporting information (S1 Text). Data from both studies is shown in Fig 3.

### Life cycle model

Adult salmonid returns to the upper Willamette River have declined 15-fold since the 1920s [31]. Following the ESA-listing for Chinook salmon in 1999, the Oregon Department of Fish and Wildlife and the National Marine Fisheries Service developed a recovery plan that emphasized the protection of remaining high-quality habitats as well as active restoration to improve degraded areas [32]. Physical habitat improvements prioritized freshwater habitat access as well as riparian and instream projects to increase habitat complexity. Water quality goals included clean (devoid of contaminants), cool, and well-aerated surface flows.

**Fig 4:**
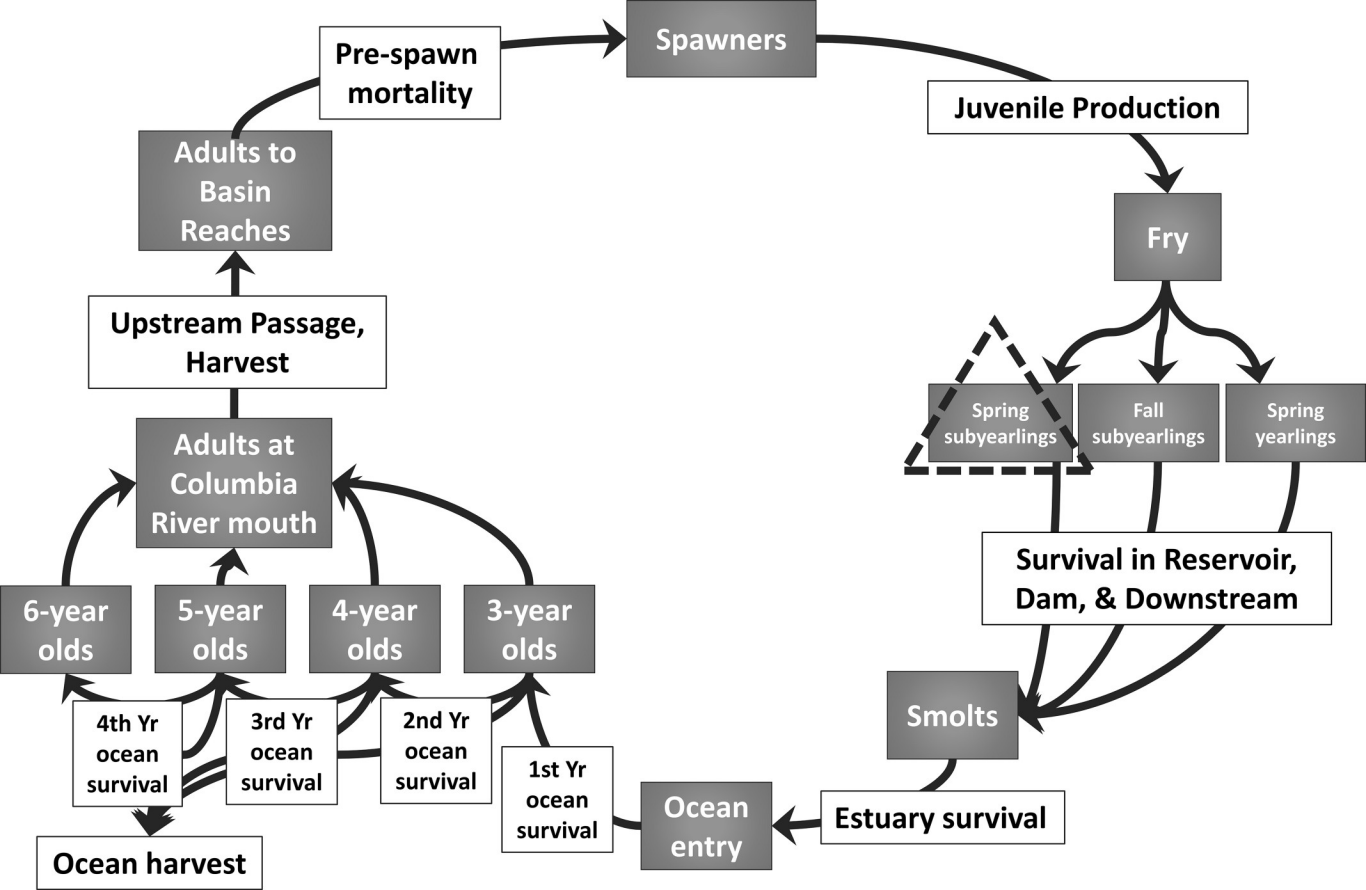
Simplified life cycle model of the McKenzie River Chinook salmon population (Upper Willamette River ESU) [18]. Gray boxes represent life stages and the white boxes describe transitions (representing survival or productivity) between life stages. The dashed triangle highlights the juvenile life-history pathway that is the focus of this study.

There are 13 federally-operated dams and reservoirs within the Willamette River Basin that are managed for flood control, recreation, irrigation, fish and wildlife habitat, and power generation. On July 11, 2008, the National Marine Fisheries Service (NMFS) concluded in a Biological Opinion that operation of the dams was likely to jeopardize the continued existence of ESA-listed fish in the Willamette Basin. In response, the U.S. Army Corps of Engineers (USACE) and regional partners developed a suite of proposed actions designed to increase Chinook salmon and steelhead population abundance. One of these actions was to evaluate dam passage scenarios through the development of individual life cycle models for ESA-listed fish populations, including four UWR Chinook populations that are directly affected by USACE dams in the McKenzie, North Santiam, South Santiam, and Middle Fork rivers [18]. The current study modifies the McKenzie River Chinook salmon life cycle model to include effects of toxic remediation in Portland Harbor (Fig 4). A summary of the life cycle model is available in Zabel et al. [18] as well as in the supporting information (S2 Text; S1 Fig). The model parameters are available in S2 Table. Briefly, the model represented physical habitat processes as well as life-history diversity and adult pre-spawn mortality through estimates of demographic parameters defining the population’s survival, productivity, and capacity. The model also represented alternative freshwater juvenile rearing tactics [22], which subdivided wild Chinook salmon into three distinct juvenile life-history trajectories based on the timing of seaward migration. These included spring subyearling, fall subyearling, and spring yearling migrants. Model parameters were derived from several sources: monitoring studies in the McKenzie River Basin and from basins nearby with Chinook salmon populations; observations from other Chinook salmon populations having similar life history characteristics; and, input from a group of regional biologists from the Oregon Department of Fish and Wildlife (ODFW), USACE, US Fish and Wildlife Service (USFWS), and the National Marine Fisheries Service’s (NMFS) West Coast Regional Office. The model was validated by comparing outputs of spawner abundance to recent observations of spawner returns to the basin.

In addition to the model’s established physical parameters, we added empirical data for chemical habitat impacts. Our analysis focused specifically on spring subyearlings, in part due to their extended residence time in the Portland Harbor area of the lower Willamette River. Previously documented chemical exposures (tissue concentrations) were evaluated in the context of adverse impacts on the near-term survival of subyearlings, as well delayed effects on growth and disease susceptibility based on present-day habitat conditions in Portland Harbor. The evidence for estimated loss is described in the results section, and summarized in Table 1.

**Table 1:** Summary of proposed effects associated with toxicant exposures, and estimated percent loss of spring subyearling population.

Scenarios	%	Supporting information for scenario and estimated percent loss of spring subyearling population (references in parentheses)
**1, West bank lethality**	50%	● Juvenile Chinook sampled on west bank show high levels of DDT, do not appear again downstream (Fig 3), and are presumed dead ● 50% of fish travel on each bank [28]
**2, Compromised immune response**	7%	● 9% delayed mortality associated with contaminants and compromised immune response [36] ● 3–10% delayed mortality from in-river contaminants and compromised immune response [37]
**3, Reduced growth**	1%	● 1.6% and 10.8% decrease in length associated with contaminant exposure [38, 39] ● 0.4% and 2.2% decreased estuary survival associated with decreased length; mean, 1.3% [40]

The primary goal of our modeling effort was to assess the potential population-scale benefits of improving chemical habitat conditions in the lower Willamette River by removing legacy contaminants, thereby increasing the survival and productivity of subyearling Chinook. To this end, we applied a combined loss estimate of outmigrating juvenile Chinook attributable to contaminant exposure to the model. This yielded an estimated percent increase in spring subyearling survival as a consequence of habitat improvement (pollution reduction) efforts associated with the future cleanup of the Portland Harbor Superfund site. The model was run prospectively for 100 years; we calculated the mean spawner abundance across the model run. This was subsequently repeated 1000 times and the mean spawner abundance values were averaged. The model was first run unmodified from the original version (Zabel et al., 2015) to represent baseline (i.e., pre-cleanup) conditions, and then run with increased survival rates for the spring subyearling subgroup as a result of legacy contaminant mitigation in the lower river. The predicted increase in spawner abundance was reported relative to baseline conditions (i.e., no chemical habitat remediation).

### Sensitivity analysis

A sensitivity analysis was also conducted to evaluate the relative influence of different model parameters, including habitat conditions and spring subyearling survival, relative to each other, on the projected spawner abundance outputs. Our purpose in conducting the global sensitivity analysis was to aid our understanding of model behavior in response to parameter perturbations, to evaluate some of the model’s assumptions, and, to examine which parameters—relative to each other—had potentially more or less influence in model responses. Our particular interest was understanding the relative influence of the parameter that determined juvenile subyearling survival in the lower river relative to the survival and capacity parameters across the other life stages. Each parameter of the sensitivity analysis was assigned a range of plausible values, which were derived from 95% confidence intervals when empirical data were available, from ranges estimated from literature values, or, where published information was not available, using input from regional ODFW, USACE, USFWS, and NMFS biologists. Parameter values were independently drawn for each individual model iteration according to a random uniform distribution. The model was run for 1000 iterations, and each run had its own set of unique parameters. Multiple regression analysis was performed on the model inputs (parameter values) and output (spawner abundance) to evaluate relative parameter influence [33, 34]. The regression coefficients were standardized (coefficient divided by standard error), and each standardized coefficient was then divided by the absolute value of the largest standardized coefficient value for comparability. The parameters used in the sensitivity analysis included: spring subyearling survival in the lower basin; adult prespawn mortality; adult fish capacity above Cougar Dam (a major dam within the McKenzie River subbasin); survival of fry below Cougar Dam; proportions of juveniles available to pass Cougar Dam; juvenile reservoir survival; proportion of fry that rear in one of three potential juvenile migrant subgroup pathways that pass through the lower river (spring subyearlings, fall subyearlings, spring yearlings); and estuary/early ocean survival [18]. Three-way proportions of juvenile life history rearing strategies (fry to the spring subyearling, fall subyearling, or spring yearling rearing pathway; in each model iteration their sum is 1) were log-ratio transformed to maintain independence (Aitchison 1997). Pre-spawn mortality is a function of the percent of wild fish on spawning grounds and late summer water temperature. Estuary/ early ocean survival is a function of PDO (Pacific decadal oscillation, an indicator of ocean conditions) in the spring (May), and Coastal upwelling in the spring and fall (Up.May, Up.Sept).

All analyses were conducted in R version 3.2.2 [35]. Map sources: Esri, Garmin, HERE, INCREMENT P, OpenStreet contributors, and the GIS community; USGS watershed boundary data set, http://nhd.usgs.gov/wbd.html. Public domain data source for map layers.

## Results

Three possible scenarios for mortality among spring subyearling UWR Chinook salmon in Portland Harbor were supported by the best available scientific information on contaminant exposure and toxicity to juvenile salmon. Data from field assessments (contaminant uptake) and controlled laboratory studies (adverse health effects) were reviewed to estimate a combined loss estimate of outmigrating juvenile Chinook salmon, with an emphasis on a subset of the priority contaminants for the Portland Harbor Superfund site (PCBs, DDTs, PAHs, and butyltins). The potential for near-term mortality within the harbor as well as delayed mortality attributable to sublethal toxicity (e.g., reduced growth, increased disease susceptibility) [11] were considered as endpoints. For the purposes of this analysis, the best available science was defined as (1) research performed on Chinook salmon; (2) contaminant exposures matched specifically to the juvenile life-stage; (3) studies addressing the adverse health effects of PCBs, DDTs, PAHs, and butyltins; and (4) environmental realism, in terms of exposure concentrations approximating present-day habitat conditions in Portland Harbor.

The resulting scenarios, summarized in Table 1, include near-term mortality of Chinook salmon navigating the west bank of the Willamette River (Scenario 1); delayed mortality of both east and west bank migrants due to immunotoxicity and a subsequent increase in disease-related losses further downstream (Scenario 2), and delayed mortality among east and west bank migrants attributable to reduced growth (Scenario 3). The evidence for each scenario of mortality is described below, as the basis for parameterizing the survival portion of the life cycle model.

### Disappearance of west bank migrants (Scenario 1)

The migration path for subyearling Chinook salmon through Portland Harbor–i.e., the east or the west bank of the river–is a key determinant of contaminant exposure. The small subyearlings travel, rest, and feed near the banks and it is unlikely they cross the deep channel of the river [22]. Thus, the east and west migrants represent different subsets of the same population that are spatially divergent in transit before remixing in the estuary below Portland Harbor. Legacy contaminants within the Portland Harbor Superfund site are not evenly distributed [24], yielding different exposure profiles for fish depending on which side of the river they travel down. For example, there is a well-characterized DDT hotspot on the west bank of Portland Harbor near the T02 sampling site (Fig 2) [24]. As shown in Fig 3, measured DDT concentrations in the tissues of juvenile Chinook salmon collected near the hotspot (∑3DDTs; site T02, 14,832 ng/g lipid) were 11- to 35-fold higher than those measured in the tissues of subyearlings from sites upstream on the east bank (Morrison Street Bridge 2005, 1,006 ng/g lipid) and west bank (Morrison Street Bridge 2013, 416 ng/g lipid; T03, 1,304 ng/g lipid) [27]. In support of demonstrating bank fidelity, east bank migrants collected at site T01, downstream of this hotspot and across the river, had relatively low levels of measurable DDTs (site T01, 1,280 ng/g lipid) [27]. Assuming the proportional distribution of larger juveniles on either side of the river channel extends to younger and smaller fish [28], we assumed that approximately 50% of the subyearling juvenile Chinook salmon travel down the west bank where they are locally exposed to high levels of DDTs in central Portland Harbor.

The available evidence suggests west bank migrants with elevated DDT concentrations do not survive the journey downriver of Portland Harbor. The divided cohort remixes at the confluence of the Willamette and Columbia Rivers, as well as where the Multnomah Channel meets the Columbia River. UWR juvenile Chinook salmon whole body composite samples were collected from ocean-ward locations in the Columbia River Estuary. Relative to DDT levels in west bank fish from the hotspot in Portland Harbor, measured tissue concentrations at Campbell Slough (1,062 ng/g lipid) and Ryan Island (1,018 ng/g lipid) were 14-fold lower [26]. This would not be expected if east and west bank migrants were evenly remixed at these estuary locations. Moreover, the pharmacokinetics of DDTs support rapid absorption (days) with slow to negligible depuration over a much longer timescale of months or years [41, 42]. As such, lower DDT levels in estuary fish were not a consequence of depuration. Growth dilution was also not explanatory, as the subyearlings grew from ~2 g in the Willamette River [28] to ~5 g at Campbell Slough and Ryan Island [43]. This almost 3-fold increase in growth would not account for the 14-fold decrease in DDT concentrations.

For the purposes of our life cycle modeling exercise and demonstrating the tool this framework provides for incorporating chemical habitat related fish loss, we made a simple assumption of zero survival of west bank migrants, corresponding to a 50% loss of all subyearling UWR Chinook salmon below Portland Harbor. This assumption comes with an acknowledgment of limited sampling below Portland Harbor to date, as well as the degree of effort required to sample for a specific population of fish amongst all the other fish from different sites and stocks. The latter is exemplified by the mere two composite samples of UWR Chinook salmon available across 5-years (2005–2009) of monthly sampling efforts and 1,200 collected juveniles in the lower Columbia River [26].

### Delayed mortality due to increased disease susceptibility (Scenario 2)

Multiple contaminants that are focal contaminants for the Portland Harbor Superfund site, including halogenated and polycyclic-aromatic hydrocarbons, and chlorinated pesticides, are known to compromise immune system function in fish and other species [44]. For salmonids specifically, controlled exposures to PAHs, PCBs, DDTs, and TBTs have been shown to decrease the antigen response as well as decrease the proliferation and viability of immune cells [45–50]. This loss of immunocompetence corresponds to an increase in disease susceptibility and higher rates of mortality when contaminant-laden juvenile salmon are exposed to environmental pathogens. The most relevant data for our purposes comes from two categories of studies, each with exposure to a similar suite of contaminants as Portland Harbor–field-collected fish from the Duwamish River (Seattle, USA) with a similar history of legacy pollution, and controlled exposure experiments. Specific to Pacific Northwest habitats, juvenile Chinook salmon immunocompetence evaluations in the form of disease-challenge studies have been conducted exposing fish with elevated contaminant levels to virulent bacterial agents *Vibrio anguillarum* and *Aeromonas salmonicida* [36, 51–53]. The resulting increased mortality relative to a low tissue contaminant level control group exposed to the same dose of pathogen was consistent in both fish from a contaminated estuary as well as hatchery fish injected with sublethal doses of contaminates [36, 53]. At moderate pathogen concentrations, 86% more contaminated estuary fish died compared to non-contaminated hatchery controls, and up to 24% excess mortality above non-contaminated controls in the fish injected with sublethal doses of contaminants. Similarly, 9% more contaminated estuary fish died than non-contaminated controls at the lower pathogen concentration (the lower pathogen concentration was not part of the injection experimental design). The discrepancy of higher mortality in the contaminated estuary fish compared to the fish injected with sublethal concentrations of contaminants may be attributed to additive or synergistic interactions among multiple chemical stressors, as described by Loge et al. (35). A parallel disease challenge experiment with high molecular weight PAH dietary exposure only (0.66 ug PAH mixture/g fish/day) was conducted using a freshwater pathogen, *A*. *salmonicida* [51]. The survival results for this study were mixed with one PAH exposure tank showing an increase in mortality relative to the non-exposed controls, with no change in mortality in the PAH exposure replication tank.

The delayed mortality in the studies above occurred over a timescale of 7–50 days. In the natural environment, pathogen exposures to outmigrating juvenile Chinook salmon are widespread and common, as evidenced from field surveys of 12 Pacific Northwest estuaries, including the Columbia River [54]. Therefore, fish traveling through Portland Harbor are exposed to immune-disrupting contaminants, including but not limited to PCBs, DDTs, PAHs, and TBTs, and are likely to subsequently encounter a pathogen in the Columbia River estuary, resulting in losses attributable to infection and disease. For our life cycle modeling, we assigned a delayed mortality rate of 7% for subyearling Chinook. This is likely a conservative estimate, below the 9% increase in mortality from the low potency disease challenge experiments described earlier [36]. In further support of this, delayed mortality from co-exposure to chemical contaminants and *V*. *anguillarium* were previously estimated to be 3–10% for juvenile salmonids migrating out of the Columbia River basin [37].

### Decreased survival due to reduced growth (Scenario 3)

First-year growth, size at migration, and size at the time of estuary and ocean transitions are critical determinants of survival for salmon. For example, extensive passive integrated transponder (PIT) tagging studies in the Columbia River Basin have shown that juvenile length at migration is a strong predictor of subsequent ocean survival [40]. The energetic costs of contaminant exposures are well known in fish and other species, in the forms of cellular detoxification, transport and sequestration, and repair [55]. Moreover, the quality and abundance of prey available to juvenile salmon in highly contaminated habitats is often poor. Therefore, toxics can have both direct and indirect effects on energy assimilation, growth, and survival, with consequences for the abundance and intrinsic growth rate of wild populations [15, 56].

Exposure to the contaminants present in Portland Harbor sediments and associated assimilations in fish tissue have previously shown to reduce growth in juvenile Chinook salmon. For example, field-collected fish from an estuary with a similar contamination of legacy pollutants (again, Duwamish River, Seattle) showed a 10.8% reduction in length compared to non-contaminated fish from their reference hatchery when the two groups were subsequently raised on a consistent diet for 84 days in clean water (34.0% increase from initial length of 91 mm vs 44.8% increase from initial length of 87 mm) [39]. Similarly, injections of PCB mixtures or contaminated sediment extracts significantly decreased juvenile growth by 2.1% and 1.1%, respectively (average: 1.6%) when the treatment groups were subsequently raised on a consistent diet for 60 days in clean water [38]. The reported growth effects were attributed to PCBs and DDTs, and not PAHs. While a PAH-contaminated diet has been shown to reduce Chinook salmon growth [57], this only occurred at concentrations well above those likely to be present in the diets of subyearlings in Portland Harbor based on previous analyses of stomach contents [27]. Less is known about reduced growth and TBT exposure; however TBTs are metabolic disruptors and reduced length has been demonstrated at higher concentrations [58, 59].

For the present analysis, reduced growth among contaminant-exposed subyearlings was linked to delayed mortality using the estuary survival potential equation, adapted from Zabel and Achord (37) (Eq 1). In the Columbia River Estuary, the average percent survival for McKenzie River Chinook salmon is approximately 66% [18]. The percent of spring subyearlings across the different life-history types that comprise the McKenzie River population ranged from 7% to 34% across seven sampling years between 2004–2013 (average 18%). Thus, a 66% survival for 18% of the population (spring subyearlings) yielded an estuarine survival parameter value of 0.1188. This parameter estimate was used to calibrate the intercept term (-2.003) when change in fish length (Δ length) was zero, as follows:

$$Estuary\;survival=\frac{\exp\left[-2.003+\left(0.0329\;x\;\Delta \;length\right)\right]}{1+\exp\left[-2.003+\left(0.0329\;x\;\Delta \;length\right)\right]}$$ (Eq 1)

Subyearlings collected from Portland Harbor range in length from 50–80 mm [27]. We therefore chose a mean length of 65 mm for the purposes of modeling. Prior studies on contaminant exposure and juvenile Chinook salmon growth were used to parameterize changes in fish length (Δ length). First, Δ length was calculated as -1.04 mm based on the 1.6% decrease in length in hatchery fish injected with a PCB mixture at environmentally relevant concentrations [38]. The second analysis used -7.02 mm as the Δ length based on a 10.8% reduction in length in Chinook salmon collected from a similarly polluted habitat [39]. Inputting these values for Δ length into Eq. 1 resulted in a respective 0.35% and 2.21% decrease in the percent of McKenzie River Chinook salmon survival in the Columbia River Estuary. For the purposes of this study a 1.0% decrease in survival, the approximate average of these values, was used to represent the impact of decreased growth for the population models.

### Modeled estimates of pollution-driven mortality at the population scale

We evaluated the extent to which chemical habitat improvements in Portland Harbor might support an increase in population abundance and, by extension, the recovery trajectory of McKenzie River Chinook salmon. This was accomplished in the established modeling framework by increasing the annual proportion of subyearling juveniles surviving the seaward migration and then returning to the McKenzie River basin to spawn.

The three considered scenarios for contaminant-driven mortality were combined on the assumption that the different categories of toxicity to individual subyearlings occur concurrently. Therefore, disruptions of immune function (7% loss) and growth (1% loss) were treated as mutually exclusive events for ease in this modeling exercise, despite an acknowledged potential for interactions between these two adverse health endpoints. The 8% combined loss estimate was halved to represent the east and west bank out-migrants. A zero survival of west bank migrants, as exemplified by the disappearance of the fish with high DDT tissue concentrations, accounted for an estimated 50% loss of spring subyearlings. The 4% loss attributed to the east bank outmigrants, as described above, was added to the 50% loss attributed to the west bank fish. Combining these east and west bank estimates resulted in a predicted 54% annual loss of spring subyearlings (Table 2).

**Table 2:** Relative percent change in McKenzie River Chinook spawner abundance predicted by percent increase in spring subyearlings; percent change is relative to baseline median predicted value for spawner abundance.

Percent increase in spring subyearlings	Relative change in spawner abundance, median (quartiles: 25^th^, 75^th^)	Extinction risk
0	0% (-44%, 123%)	0.24 (moderate/high)
54	25% (-56%, 177%)	0.08 (low/moderate)

Removing pollution stress from Portland Harbor via sediment remediation and other cleanup measures would presumably prevent these losses. A 54% increase in subyearling survival to the population model yielded an estimated increase in mean spawner abundance of 25% [interquartile range (IQR) (25^th^, 75^th^); -56%, 177%]. The 5^th^ and 95^th^ quantiles were -80% and 499%, respectively. The baseline model, run with no change to the spring subyearling survival, estimated the increase to be 0% (IQR; -44%, 123%). The 5^th^ and 95^th^ quantiles for the baseline model were -84% and 394%, respectively. This large relative range, even when modification of spring subyearling survival was not incorporated into the model, can be attributed to inherent fluctuations in population sustainability and trends, including degree of harvest, environmental conditions (habitat quality–such as alteration in temperature, sedimentation, stream flows, etc), and effects of artificial propagation [60].

The risk of extinction for the McKenzie River population was calculated from the proportion of the n = 1,000 model runs yielding a spawner abundance projection that was lower than a quasi-extinction threshold of 250 adult fish [61]. The baseline model runs–i.e., without a contaminant exposure and toxicity component–reported an extinction risk of 0.24. This probability lies on the high risk end of the defined category of relatively moderate (0.05–0.25) risk for extinction in 100 years [18]. A 54% increase in spring subyearling survival lowered the projected extinction risk to 0.08 over the next century. Our modeling therefore suggests that a cleanup of pollutants (including, PCBs and DDTs) in Portland Harbor could shift the conservation outlook for McKenzie River Chinook salmon, from a high risk of extinction to a lower risk.

### Parameter relative influence

**Fig 5:**
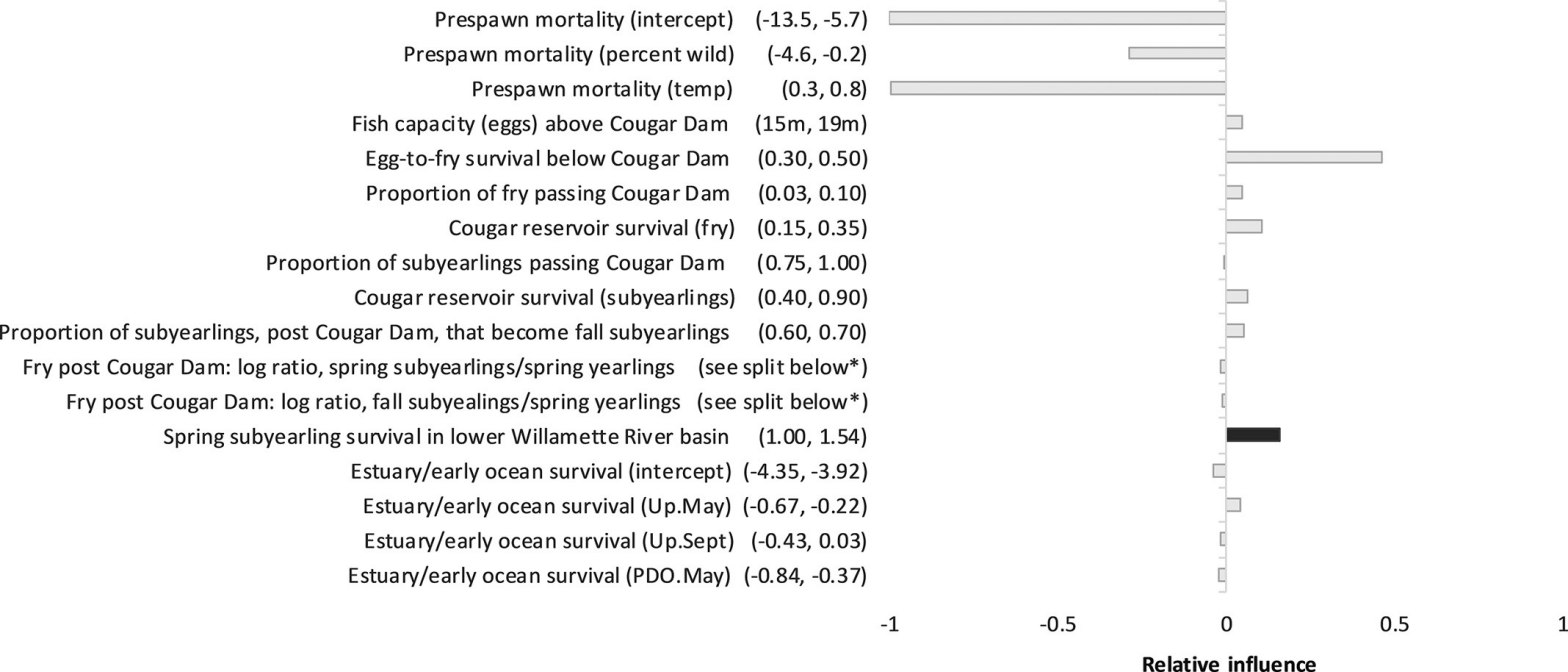
Sensitivity analysis on median predicted value for spawner abundance conducted on select parameters in the McKenzie Chinook salmon life cycle model. Dark bar indicates influence of increased spring subyearling survival as estimated by removal of toxic insults; parameter ranges are in parentheses. Parameter ranges for juvenile subgroups: spring subyearlings (0.10, 0.33); fall subyearlings (0.40, 0.65); and yearlings (0.10, 0.30). Abbreviations: m (million), PDO (Pacific decadal oscillation, an indicator of ocean conditions), Up.May (Coastal upwelling indices, May), and Up.Sept (Coastal upwelling indices, September).

To evaluate the relative influence of chemical habitat improvements (pollution reduction or removal) with respect to other model parameters related to Chinook salmon habitat and recovery, a sensitivity analysis was performed. The model was not run in a prospective manner as depicted in the results for Table 2, but rather as a way to explore model behavior in response to simultaneously and independently perturbing selected parameters. Results of the sensitivity analysis indicate which among a set of parameters, relative to each other, have potentially more or less influence in the life cycle model. Spring subyearling survival was included with the parameter value range of 1.00 (no effect) to 1.54 (representing 54% increase in juvenile salmon abundance). Actions that reduce adult prespawn mortality were the most influential. However, contaminant removal to improve spring subyearling survival was more influential than parameters associated with early ocean survival and proportion of fry passing Cougar Dam (Fig 5).

## Discussion

Major societal investments to conserve and recover Pacific salmon and steelhead species have been ongoing for decades, and will continue for the foreseeable future. Much of this work has focused on physical habitat restoration, and these efforts have not been sufficient to restore population abundances to the point where a threatened or endangered species has been delisted from the ESA. Accordingly, the natural resource management community is increasingly considering other types of habitat actions, including improvements to water and sediment quality. However, the relative costs and benefits of physical vs. chemical restoration, particularly at the population-scale, remain poorly understood. Here we have used an established life cycle model for UWR spring Chinook salmon to estimate the consequences of pollution mitigation in Portland Harbor, in terms of improving subyearling survival and its effects on the number of adults that return to spawn each year in the McKenzie River watershed. Our results indicate that current and future cleanup efforts at the Portland Harbor Superfund site will likely benefit the recovery of this Chinook population. Our model outputs suggest a ~20% increase in spawner abundance over current estimates as a result of a simulated removal in legacy contaminant exposure in Portland Harbor.

Our approach provides an initial framework for incorporating toxic chemicals into the larger picture of endangered species conservation. Nevertheless, existing uncertainties (discussed below) constrain the interpretation of our current findings. Our goal was to connect previous field and laboratory studies for chemical exposure and adverse health outcomes in juvenile Chinook salmon to an established framework for modeling population recovery in response to habitat improvement. In some cases, the best available science was incomplete (reconnaissance sampling for outmigrating juveniles in the Willamette River) or based on a relatively older literature (controlled toxicity studies). Where information was lacking, we made simple and transparent assumptions. In the future, these assumptions can be directly addressed via 1) more comprehensive and targeted field surveys of contaminants and adverse health indicators in juvenile Chinook salmon from Portland Harbor, and 2) laboratory toxicity studies matched to documented, present-day exposure conditions.

Consistent with previous studies [16, 62], our model simulation indicates the importance of first-year survival for Chinook salmon population abundance. We focused on spring subyearlings because this life-history type spends more time residing and feeding in the lower Willamette River, to achieve necessary growth prior to entering the Columbia River estuary. Delayed mortality has previously been demonstrated for juvenile Chinook salmon that spend time in habitats with a history of industrial pollution [39]. In support of this all sites sampled within Portland Harbor (T01-T03) showed accumulated juvenile Chinook salmon tissue concentrations of PCBs above the threshold for adverse sublethal effects (2,400 ng/g lipid) (Fig 3) [63]. A more recent analysis of smolt-to-adult return (SAR) rates for 230 million hatchery-reared Chinook salmon released between 1972–2008 in Puget Sound found ~ 40% lower survival rates for juvenile Chinook salmon outmigrating through and rearing in contaminated estuaries (SAR = 0.48%) versus relatively clean estuaries (SAR = 0.87%) [64]. Also, the available evidence suggests that west bank migrants do not survive exposures to DDTs and possibly other adverse habitat factors (contaminants or otherwise) on the west bank. Prior sampling of juvenile fish below Portland Harbor included over 1,200 fish, collected monthly over a five-year period. Nevertheless, this may not have been intensive enough to capture the heavily contaminated UWR Chinook salmon. Similarly, since these were composite samples, if a couple of very contaminated fish were included with those less contaminated, the effect would be diluted. Nonetheless, samples within Portland Harbor (T02) demonstrated DDT levels above the threshold concentration associated with no or low biological effects [600 ng/g converted to 6,000 ng/g lipid; much of the toxicity data used in development of the threshold were laboratory reared, which have 8–10% lipids [63]] reinforcing the likely lethality of these juveniles [65].

Our literature-based estimates for delayed mortality among UWR Chinook salmon juveniles are likely conservative, for several reasons. First, we did not include losses to older and larger juvenile subgroups Rather, our estimate only included the spring subyearlings which represent an estimated 18% of the McKenzie River population [21]. While dietary exposures may be lower for the other juvenile subgroups as a consequence of a shorter residence time in Portland Harbor, contaminants could nevertheless adversely affect the health of these fish. Second, we did not address other classes of legacy toxics that are known to persist in Lower Willamette sediments. Metals such as barium and manganese are fairly common in sediments contaminated by historical industrial practices, including Portland Harbor. Under natural exposure conditions, UWR subyearlings are likely exposed to a larger and more complex set of contaminants than evaluated in this study, thereby exacerbating inhibitory effects on subsequent growth and immune function. Single chemical-based risk assessments may underestimate impacts from exposures to toxic chemicals due to mixture additivity or synergism [66, 67]. Lastly, the tissue levels of PCBs, DDTs, PAHs, and TBTs used here represent an exposure snapshot, and it is possible that the subyearling Chinook sampled would have resided in Portland Harbor for longer periods (days or weeks), thereby increasing persistent pollutant bioaccumulation.

Our estimates of delayed mortality would invariably be refined with additional exposure and sublethal toxicity information. Existing data from field-collected fish in Portland Harbor represent the best available information, and yet are limited in terms of the number of Chinook sampled and the spatial coverage above, within, and below the contaminated areas of the lower Willamette River. The exposure profiles for PCBs, DDTs, PAHs, and TBTs are also dated, and will become increasingly unrepresentative as sediment remediation and other cleanup activities progress in the coming years. Future collections of outmigrating subyearlings should be expanded in space and time, in addition to the established T01-T03 locations. Chemical analyses of the diets and tissues of Chinook salmon should also be expanded to include other toxicants of concern, as well as consider composition and abundance of their diet as an indicator of impacts of contaminants on their prey source. This will give a more complete picture of residence time in the lower Willamette River, as well as cumulative dietary exposure to a broader range of POPs. This will also directly evaluate our preliminary assumption that west bank migrants do not survive due to adverse habitat factors such as exposure to DDTs and possibly other contaminants.

The toxicology literature for many focal contaminants in Portland Harbor sediments is also relatively old, commensurate with the DDT and PCB bans in the 1970s. Nevertheless, these chemicals persist in salmon habitats. As we have shown here, they will likely continue to be a limiting factor for the recovery ESA-listed UWR Chinook salmon until cleanup activities are completed. Future sampling can build on the older scientific literature by applying emerging technologies, including genomics, bioinformatics, and pathway analysis. The field of biomarker development is evolving rapidly, yielding new insights into mechanisms of toxicity (growth, immunomodulation, etc.) as well as new molecular indicators that are phenotypically anchored to injury (reduced growth, disease susceptibility, etc.) and delayed mortality. The direct sequencing of the subyearling Chinook salmon transcriptome and subsequent identification of differentially expressed genes known to be involved in molecular initiating events such as aryl hydrocarbon receptor (AHR) activation, as well as physiological pathways governing growth, pathogen resistance, and adaptation, would be informative [17]. There will also be a role for other methods in future sampling, including estimates of growth from otolith and insulin-like growth factor (IGF) measurements. For example, there is some preliminary but suggestive evidence that Portland Harbor west bank migrants have a modified growth rate relative to east bank fish [data unpublished, methods from [68]].

In conclusion, we have used a life cycle modeling approach to estimate the relative benefits of chemical habitat restoration for an ESA-listed salmon population. Our results suggest that future cleanup of Portland Harbor, and subsequent reductions in lethal and sublethal toxicity to subyearling juveniles, has the potential to improve the viability of UWR spring Chinook salmon population by increasing subyearling survival and, by extension, adult spawner abundance. Our methods are transferrable to other species that are vulnerable to habitat pollution. Future field assessments, in coordination with the ongoing cleanup of legacy contamination in Portland Harbor, will offer opportunities to empirically test key assumptions in our current study–i.e., that contaminant exposures will decrease, subyearling health will improve, delayed mortality will be reduced, and the subyearling cohort contribution to adult spawner abundance will increase. This framework provides a useful albeit initial tool to evaluate each of several management actions related to physical, biological, and chemical habitat restoration.

## Supporting information

S1 Fig The McKenzie River system. (PDF)

S1 Table Mean percent lipids and concentrations of PCBs, DDTs, tributyltin ion, and PAHs in outmigrating juvenile Chinook salmon collected from three sampling sites within Portland Harbor (T01-T03), two sites downstream (Ryan Island and Campbell Slough), and two sites upstream (T04 and Morrison St Bridge). (PDF)

S2 Table Parameters included in the life cycle model. (PDF)

S1 Text Tissue residue values from Portland Harbor, analytic methods of chemical analysis. (PDF)

S2 Text McKenzie River spring-run Chinook salmon life cycle model description. (PDF)
