# Supplementary material for: Legacy habitat contamination as a limiting factor for Chinook salmon recovery in the Willamette Basin, Oregon, USA
Source: PLoS One. 2019 Mar 22;14(3):e0214399. doi: 10.1371/journal.pone.0214399 (PMC6430382; doi:10.1371/journal.pone.0214399)

S1 Figure. The McKenzie River system, a major tributary to the Willamette River, Oregon, USA (inset), is home to the McKenzie River spring-run Chinook salmon population.

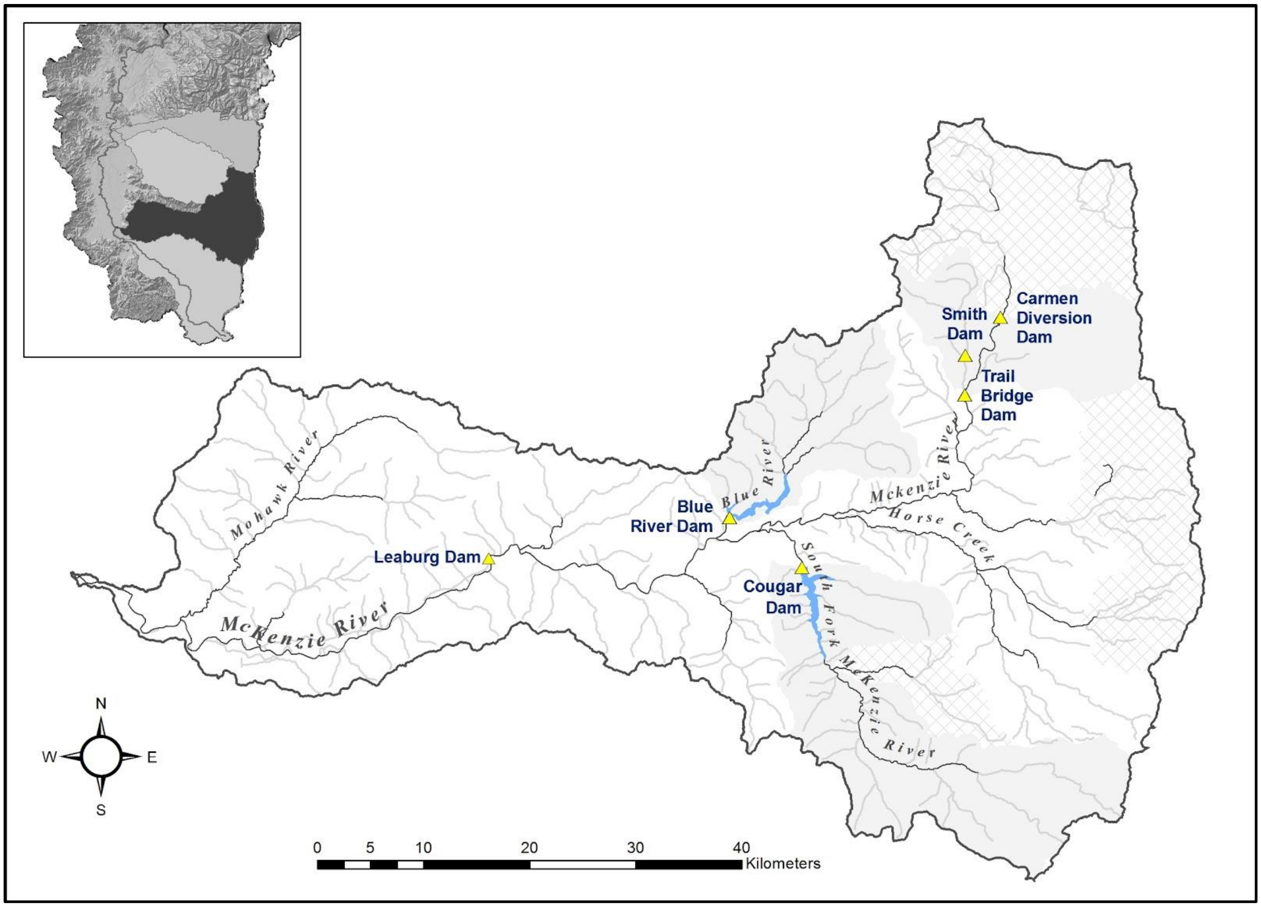

Supplement: S1 Fig — (PDF) [file pone.0214399.s001.pdf]
