# Supplementary material for: Legacy habitat contamination as a limiting factor for Chinook salmon recovery in the Willamette Basin, Oregon, USA
Source: PLoS One. 2019 Mar 22;14(3):e0214399. doi: 10.1371/journal.pone.0214399 (PMC6430382; doi:10.1371/journal.pone.0214399)
Supplement: S1 Table — (PDF) [file pone.0214399.s002.pdf]

S1 Table. Mean percent lipids and concentrations of PCBs, DDTs, tributyltin ion, and PAHs in outmigrating juvenile Chinook salmon collected from three sampling sites within Portland Harbor (T01-T03), two sites downstream (Ryan Island and Campbell Slough), and two sites upstream (T04 and Morrison St Bridge).

Sampling included whole body and stomach content composite samples (a: Johnson et al. 2013 [1]; b: DIVER, 2017 [2]; c: unpublished data collected by L. Johnson [3]). Whole body samples were composites minus the stomach contents. Values in parentheses are number of composite samples and standard deviation. NA=not applicable.

	% Lipids (whole body)	PCBs[e] (whole body) ng/g lipid	DDTs[e] (whole body) ng/g lipid	Tributyltin ion (whole body) ng/g ww	PAH (stomach contents) ng/g ww
**Ryan Island[a]**	1.6 (1, NA)	1457 (1, NA)	1019 (1, NA)	Not measured	Not measured
**Campbell Slough[a]**	1.5 (1, NA)	2514 (1, NA)	1069 (1, NA)	Not measured	43 (3, 23)
**T01[b]**	1.5 (3, 0.06)	9801 (3, 7852)	1280 (3, 500)	2.2 (3, 0.4)	299 (2, 145)
**T02[b]**	1.7 (3, 0.06)	5900 (3, 244)	14832 (3, 471)	4.1 (2, 0)[d]	1364 (2, 1545)
**T03[b]**	1.8 (3, 0.06)	11352 (3, 3636)	1304 (3, 290)	1.7 (3, 0.4)[d]	95.5 (1, NA)
**Morrison St Bridge 2013[c]**	2.2 (4, 0.5)	1333 (4, 730)	416 (4, 84)	Not measured	155 (1, NA)
**Morrison St Bridge 2005[a]**	2.0 (4, 0.6)	1643 (4, 681)	1006 (4, 516)	Not measured	399 (7, 316)
**T04[b]**	1.8 (3, 0.06)	940 (3, 256)	492 (3, 103)	0.4 (3, 0.04)	87 (1, NA)

a,c: Analysis by NOAA Northwest Fisheries Science Center (Seattle, Washington, USA). Stomach content composite samples were analyzed for PAHs [24 total: naphthalene, 1-methylnaphthalene, 2-methylnaphthalene, biphenyl, 2,6-dimethylnaphthalene, acenaphthylene, 2,3,5-trimethylnaphthalene, acenaphthene, fluorene, retene, phenanthrene, 1-methylphenanthrene, anthracene, fluoranthene, pyrene, chrysene + triphenylene (coelute), benzo[*a*]pyrene, benzo[*e*]pyrene, perylene, dibenz[*a*,*c*+*a*,*h*]anthracene (colelute), benzo[*b*]fluoranthene, benzo[*j*+*k*]fluoranthene (coelute), indeno [1,2,3-*cd*]pyrene, benzo[*g*,*h*,*i*]perylene] [4].

b: Analysis by Axys Analytical Services, Ltd. (Sydney, B.C., Canada) and Columbia Analytical Services (Kelso, Washington, USA). Whole body (minus stomach) contaminant concentrations include: butyltins (butyltin ion, dibutyltin ion, tributyltin ion, and tetrabutyltin). Stomach content composite samples were analyzed for PAHs [18 total: naphthalene, 2-methylnaphthalene, acenaphthene, acenaphthylene, anthracene, fluorene, phenanthrene, dibenz(a,h)anthracene, benz(a)anthracene, benzo(a)pyrene, benzo(b)fluoranthene, benzo(g,h,i)perylene, benzo(k)fluoranthene, benzo(b+j)fluoranthene (coelute), chrysene, fluoranthene, indeno[1,2,3-*cd*]pyrene, pyrene] [2].

d: Both samples from site T02, and one sample from site T04, also had detectable monobutyltin ion. T02: 2.6 and 4.6 ng/g ww, and T03: 5.4 ng/g ww. No other samples had detectable monobutyltin ion, dibutyltin ion, or tetrabutyltin ion.

e: For consistency across studies, PCBs reported as ∑17PCBs*2 [Morrison Street Bridge and two sampling events below the Columbia-Willamette confluence: PCBs 18, 28, 44, 52, 95, 101 (co-elution, 90), 105, 118, 128, 138 (163,164), 153 (132), 170, 180, 187 (159, 182), 195, 206, 209 [4]; T01-T04: PCBs 18 (co-elution, 30), 28 (20), 44 (47, 65), 52, 95 (93, 98, 100, 102), 101 (90, 113), 105, 118, 128 (166), 138 (129, 160, 163), 153 (168), 170, 180 (193), 187, 195, 206, 209 [2]]. DDTs reported as ∑3DDTs (p,p'-DDD, p,p'-DDE, p,p'-DDT)

References

1. Johnson LL, Anulacion BF, Arkoosh M, Olson OP, Sloan CA, Sol SY, et al. Persistent organic pollutants in juvenile Chinook salmon in the Columbia River basin: implications for stock recovery. Transactions of the American Fisheries Society. 2013;142(1):21-40.
2. [Data Integration Visualization Exploration and Reporting] Web Application, National Oceanic and Atmospheric Administration. Region: Northwest, Collection study name: Portland Harbor Round 2A Juvenile Chinook 2005. Data can be queried and downloaded at: https://www.diver.orr.noaa.gov/web/guest/diver-explorer?siteid=2&sqid=663 [Internet]. 2017.
3. Johnson LL, Ylitalo GM. Unpublished data, sample data at Morrison Bridge in Portland, Oregon, USA. 2013.
4. Sloan CA, Brown DW, Pearce RW, Boyer RH, Bolton JL, Burrows DG, et al. Extraction, Cleanup, and Gas Chromatography/Mass Spectrometry Analysis of Sediments and Tissues for Organic Contaminants. NMFS-NWFSC-59. U.S. Dept. Commer., NOAA Tech. Memo. Seattle, Washington, USA. 2004.
