# Supplementary material for: Legacy habitat contamination as a limiting factor for Chinook salmon recovery in the Willamette Basin, Oregon, USA
Source: PLoS One. 2019 Mar 22;14(3):e0214399. doi: 10.1371/journal.pone.0214399 (PMC6430382; doi:10.1371/journal.pone.0214399)
Supplement: S2 Table — (PDF) [file pone.0214399.s003.pdf]

S2 Table. Parameters of the McKenzie River spring-run Chinook salmon population life cycle model [1].

Parameter	Description	Below Leaburg Dam (Reach A)	Above Leaburg dam, below Cougar Dam (Reach B)	Above Cougar Dam and reservoir (Reach C)
bbox	Mainstem upstream adult *en route* migration survival, after harvest has been accounted for	0.716		
NOR.total.initial	Initializing values of natural origin (NOR) spawners to reaches; 2007-2011 geomean of estimated spawners, n = 1693, split by reach (0.118, to reach A, of the 0.882, 0.72 to B, and 0.28 trucked to C	199	1075	418
HOR.total.initial	Number of hatchery-origin (HOR) spawners used to initialize the model	300	500	800
NOR.broodstock	Number of NOR fish used for hatchery broodstock, collected from reach B	---	200	---
HOR.broodstock	Number of HOR fish used for hatchery broodstock,	---	400	---

Parameter	Description	Below Leaburg Dam (Reach A)	Above Leaburg dam, below Cougar Dam (Reach B)	Above Cougar Dam and reservoir (Reach C)
	collected from reach B			
splits.HOR.strays	Distribution of HOR on spawning grounds after 82% return to hatchery rack	0.01908	0.1158624	0.0450576
max.spawned	Maximum number of fish (NOR + HOR) used for broodstock	600		
hatchery.yearlings.released	Function that determines the number of hatchery yearlings released	Fluctuates annually, hockey stock function estimates yearlings by multiplying hatchery broodstock by 1600 up to the max.spawned if the maximum are collected for broodstock, thereafter yearlings released is drawn from a triangular distribution (min = 750,000, mean = 800,000, max = 850,000, sd = 50,000)		
HOR.outplant.func	Number of HOR trucked above Cougar Dam (CGR) to spawn in the wild	---	Fluctuates annually, collected at 0.28 rate at CGR, up to HOR.max = 600, decreases linearly with increasing NOR collected at Cougar Dam and trucked above (0 HOR when NOR >= 800)	
psm	Prior to spawning, fish mortality as a function of water temperature and proportion of hatchery origin fish on spawning grounds (pHOS)	logit(PSM) = -9.5789 + 05492*T – 2.39*pHOS		
eggs	Spawners to eggs transition with a Beverton-Holt (BH) production function,	eggs = (Spawners*p1)/(1 + (p1/c1.egg)*Spawners)		

Parameter	Description	Below Leaburg Dam (Reach A)	Above Leaburg dam, below Cougar Dam (Reach B)	Above Cougar Dam and reservoir (Reach C)
	dependent on egg capacity and egg-fry survival			
c1.egg	Egg capacity used in the BH function, fluctuates annually and is drawn from a triangular distribution (min, mean, max, sd set for each reach)	Min = 250,000<br>Mean = 500,000<br>Max = 600,000<br>Sd = 50,000	Min = 2,000,000<br>Mean = 8,000,000<br>Max = 15,000,000<br>Sd = 250,000	Min = 4,000,000<br>Mean = 17,000,000<br>Max = 22,000,000<br>Sd = 500,000
p1	Productivity (eggs per fish) used in the BH function; fecundity of 5000 eggs/female for males present by adjusting by 0.45 multiplier (2.22 fish per redd)	2,250		
discount.NOR	Maximum domestication discount applied to progeny of NOR to account for legacy hatchery effects; linearly decreases to 0 with proportionate natural influence approaching 1.0	0.20		
discount.HOR	Maximum domestication discount applied to	0.50		

**Parameter**	**Description**	**Below Leaburg Dam (Reach A)**	**Above Leaburg dam, below Cougar Dam (Reach B)**	**Above Cougar Dam and reservoir (Reach C)**
	progeny of HOR to account for hatchery domestication effects; linearly decreases to 0 with proportionate natural influence approaching 1.0			
egg.fry.survival	Egg to fry survival	0.35	Fluctuates annually due to total dissolved gas effects, dependent on CGR alternative	0.575
fry.subyearling.split	Fry available to pass dam	---	---	Fluctuates annually, dependent on CGR alternative
fry.subyearling.reservoir.survival	Fry to subyearling CGR reservoir survival	---	---	0.20
fry.splits	Life history rearing pathway of fry	Spring subyearling = 0.41<br>Fall subyearling = 0.304<br>Spring yearling = 0.286	Spring subyearling = 0.41<br>Fall subyearling = 0.304<br>Spring yearling = 0.286	spring subyearling = 0.10<br>fall subyearling = 0.60<br>spring yearling = 0.30
subyearling.yearling.reservoir.split	Subyearlings available to pass CGR	---	---	Dependent on CGR alternative
subyearling.splits	Life history rearing pathway of subyearlings that pass CGR	---	---	Fall subyearling = 0.60<br>Spring yearling = 0.40
subyearling.yearling.reservoir.survival	Survival of subyearlings to	---	---	0.65

**Parameter**	**Description**	**Below Leaburg Dam (Reach A)**	**Above Leaburg dam, below Cougar Dam (Reach B)**	**Above Cougar Dam and reservoir (Reach C)**
	yearling stage in CGR reservoir			
DPE	Proportion of fish passing CGR that are available to pass it (dam passage efficiency)	---	---	Fluctuates yearly and juvenile stage-dependent, dependent on CGR alternative
fry.fbw, subyearling.fbw, yearling.fbw	Parameters associated with mortality by life history stage type determining survival passage by CGR	---	---	Fluctuates yearly and juvenile stage-dependent, dependent on CGR alternative
TDG.Juv	Mortality associated with total dissolved gas exposure of juveniles passing past CGR	---	---	Fluctuates yearly, dependent on CGR alternative
TDG.alevin	Mortality of in-gravel juveniles associated with total dissolved gas exposure prior to swim up, below CGR only	---	---	Fluctuates yearly, dependent on CGR alternative
subbasin.JOM.survival	Survival in the McKenzie basin, prior to smolting stage	spring subyearling = 0.225<br>fall subyearling = 0.175<br>spring yearling = 0.14	spring subyearling = 0.275<br>fall subyearling = 0.225<br>spring yearling = 0.17	spring subyearling = 0.225<br>fall subyearling = 0.175<br>spring yearling = 0.14
JOM.to.rivermigrant.survival	Mainstem Willamette Survival	spring subyearling = 0.10	spring subyearling = 0.175	spring subyearling = 0.15

**Parameter**	**Description**	**Below Leaburg Dam (Reach A)**	**Above Leaburg dam, below Cougar Dam (Reach B)**	**Above Cougar Dam and reservoir (Reach C)**
	from the McKenzie to below Willamette Falls	fall subyearling = 0.40<br>spring yearling = 0.525	fall subyearling = 0.40<br>spring yearling = 0.525	fall subyearling = 0.375<br>spring yearling = 0.575
hatchery.JOM.to.rivermigrant.survival	Survival of hatchery smolts to below Willamette Falls	0.825		
s3	Survival in ocean entry year; stochastic in start year of inputs to function and sampled from standard error of model fit	logit(S3) = -4.13 – 0.44*Up.May – 0.20*Up.Sept – 0.606*PDO.May		
s3.fac	s3 multiplier; s3 was estimated using HOR, multiplier aligned s3 to account for higher ocean survival	2.0		
So	Survival in the ocean after the first year	0.80		
bX	Proportion of fish that by age that mature and return at X age (3, 4, 5, or 6 yr old)	b3 = 0.002<br>b4 = 0.31<br>b5 = 0.88<br>b6 = 1.0		
ocean.harv.Xyrolds	Ocean harvest rates, by age	Age 3 = 0.002<br>Age 4 = 0.09<br>Age 5 = 0.09		
river.harv.NOR	River harvest rate of NOR	0.097		

Parameter	Description	Below Leaburg Dam (Reach A)	Above Leaburg dam, below Cougar Dam (Reach B)	Above Cougar Dam and reservoir (Reach C)
river.harv.HOR.Xyrolds	River harvest rate of HOR, by age	Age 3 = 0.002<br>Age 4 = 0.31<br>Age 5 = 0.88<br>Age 6 = 1.0		

Reference

1. Zabel RW, Myers J, Chittaro P, Jorgensen J. Viable Salmonid Population (VSP) modeling of Willamette River spring Chinook and steelhead populations. Appendix C: Detailed biological analysis, In Willamette Valley projects configuration/operation plan (COP) Phase II report, U.S. Army Corps of Engineers, Portland District, Portland, OR. [Main report: http://pweb.crohms.org/tmt/documents/FPOM/2010/Willamette_Coordination/Main%20Rpt%20COP%20II_Final_29Oct15.pdf]. 2015.
