# Supplementary material for: Legacy habitat contamination as a limiting factor for Chinook salmon recovery in the Willamette Basin, Oregon, USA
Source: PLoS One. 2019 Mar 22;14(3):e0214399. doi: 10.1371/journal.pone.0214399 (PMC6430382; doi:10.1371/journal.pone.0214399)
Supplement: S1 Text — (PDF) [file pone.0214399.s004.pdf]

S1 Text. Tissue residue values from Portland Harbor, analytic methods of chemical analysis [1-4].

The Lower Willamette Group

Chinook Salmon whole fish bodies with the stomach contents removed were homogenized by Columbia Analytical Services (Kelso, Washington, USA) and extracted for percent lipids, six DDTs (o,p'-DDD; o,p'-DDE; o,p'-DDT; p,p'-DDD; p,p'-DDE; p,p'-DDT), and four butyltins (butyltin ion, dibutyltin ion, tributyltin ion, and tetrabutyltin). For DDTs, samples were extracted with dichloromethane using a Soxhlet extraction, followed by gel permeation chromatography and Florisil column cleanup procedures. The sample extracts were analyzed by gas chromatography/ electron capture detection. For butyltins, samples were extracted using tropolone in dichloromethane, followed by derivatization and analysis by gas chromatography/flame photometric detection. Lipids were measured gravimetrically following Soxhlet extraction in dichloromethane. PCB and PAH analysis was performed by Axys Analytical Services, Ltd. (Sydney, B.C., Canada). Following a Soxhlet extraction in dichloromethane, whole body composites (gutted) for PCB analysis were cleaned using acid and acid/base silica gels followed by 1% deactivated basic Alumina and Florisil. Soxhlet extraction was used for the stomach content composite samples, followed by gel permeation chromatography for lipid removal, and further isolation of target compounds with silica gel. Samples were analyzed for PAHs [18 total: naphthalene, 2-methylnaphthalene, acenaphthene, acenaphthylene, anthracene, fluorene, phenanthrene, dibenz[*a*,*h*]anthracene, benz[*a*]anthracene, benzo[*a*]pyrene, benzo[*b*]fluoranthene, benzo[*g*,*h*,*i*]perylene, benzo[k]fluoranthene, benzo[*b*+*j*]fluoranthene (coelute), chrysene, fluoranthene, indeno[1,2,3-*cd*]pyrene, pyrene]. The PCB and PAH sample extracts, individually, were analyzed with high-resolution gas

chromatography/ high-resolution mass spectrometry. All sampling and analysis was conducted under a U.S. Environmental Protection Agency approved quality assurance project plan [5]. All generated data was determined to be "Category 1 data" defined as "[There is] sufficient information on these data sets to confidently verify that the data, along with associated data qualifiers, accurately represent chemical concentrations present at the time of sampling."

Lower Columbia River Estuary Partnership

Chinook salmon bodies with stomachs removed were extracted with dichloromethane, using an accelerated solvent extractor. The sample extracts were precleaned on an alumina–silica column, and then further cleaned up using size-exclusion liquid chromatography. The sample extracts were analyzed by low-resolution gas chromatography/mass spectrometry. Measured concentrations in whole fish bodies with the stomach contents removed included 45 PCBs (PCBs 17, 18, 28, 31, 33, 44, 49, 52, 66, 70, 74, 82, 87, 95, 99, 101/90, 105, 110, 118, 128, 138/163/164, 149, 151, 153/132, 156, 158, 170/190, 171, 177, 180, 183, 187, 191, 194, 195, 199, 205, 206, 208, and 209) and six DDTs (as listed above). Stomach content composite samples were analyzed for 24 PAHs [naphthalene, 1-methylnaphthalene, 2-methylnaphthalene, biphenyl, 2,6-dimethylnaphthalene, acenaphthylene, 2,3,5-trimethylnaphthalene, acenaphthene, fluorene, retene, phenanthrene, 1-methylphenanthrene, anthracene, fluoranthene, pyrene, chrysene + triphenylene (coelute), benzo[*a*]pyrene, benzo[*e*]pyrene, perylene, dibenz[*a,c+a,h*]anthracene (coelute), benzo[*b*]fluoranthene, benzo[*j+k*]fluoranthene (coelute), indeno[1,2,3-*cd*]pyrene, benzo[*g,h,i*]perylene] using analytical methods as described above for whole bodies. Lipids were measured gravimetrically following extraction in dichloromethane. Laboratory used

performance-based methods for quality control procedures. Analytical methods are further described in Sloan et al. [2].

Considerations in the use of data from separate labs

It should be noted the data evaluated for this project are from separate labs using different methods for sample preparation and quantification, as well as quality control procedures. For consistency across studies, PCBs were reported as ∑17PCBs*2 [Morrison Street Bridge and two sampling events below the Columbia-Willamette confluence: PCBs 18, 28, 44, 52, 95, 101 (co-elution, 90), 105, 118, 128, 138 (163,164), 153 (132), 170, 180, 187 (159, 182), 195, 206, 209 [3]; T01-T04: PCBs 18 (co-elution, 30), 28 (20), 44 (47, 65), 52, 95, 101 (90, 113), 105, 118, 128 (166), 138 (129, 160, 163), 153 (168), 170, 180 (193), 187, 195, 206, 209 [4]]. This formula provides a good estimate of the total PCBs in a typical environmental sample of sediments, or animals feeding on lower trophic levels, where a mixture of Aroclors 1254 and 1260 is the predominant pattern [6, 7]. Also, DDTs were reported as ∑3DDTs (p,p'-DDD, p,p'-DDE, p,p-DDT), which represent > 80% of the DDTs present [4]. Most target analytes were above the limit of detection, with the exception of p,p'-DDT in the Campbell Slough composite, and PCBs 195, 206, and 209 in most composites from Morrison Street Bridge, and the composites from Campbell Slough and Ryan Island. A value of half the detection limit was assigned to estimate these target analytes. Evaluations of individual-based toxicity estimates based on data from previous field assessments and controlled laboratory studies were matched by individual PAH compounds to the extent possible.

References

1. U.S. EPA. Environmental Protection Agency. Portland Harbor Superfund Site. https://yosemite.epa.gov/r10/cleanup.nsf/ph/portland+harbor+superfund+site. 2016.
2. Sloan CA, Anulacion BF, Baugh KA, Bolton JL, Boyd D, Boyer RH, et al. Northwest Fisheries Science Center's analyses of tissue, sediment, and water samples for organic contaminants by gas chromatography/mass spectrometry and analyses of tissue for lipid classes by thin layer chromatography/flame ionization detection. NMFS-NWFSC-125. U.S. Dept. Commer., NOAA Tech. Memo. Seattle, Washington, USA. 2014.
3. Sloan CA, Brown DW, Pearce RW, Boyer RH, Bolton JL, Burrows DG, et al. Extraction, Cleanup, and Gas Chromatography/Mass Spectrometry Analysis of Sediments and Tissues for Organic Contaminants. NMFS-NWFSC-59. U.S. Dept. Commer., NOAA Tech. Memo. Seattle, Washington, USA. 2004.
4. [Data Integration Visualization Exploration and Reporting] Web Application, National Oceanic and Atmospheric Administration. Region: Northwest, Collection study name: Portland Harbor Round 2A Juvenile Chinook 2005. Data can be queried and downloaded at: https://www.diver.orr.noaa.gov/web/guest/diver-explorer?siteid=2&sqid=663 [Internet]. 2017.
5. U.S. EPA. Portland Harbor Remedial Investigation and Feasibility Study. Remedial Investigation Report. Final. February 8, 2016. Found at https://semspub.epa.gov/src/collection/10/SC34260. Superfund Site: Portland Harbor (EPA ID: ORSFN1002155), Collection ID: 34260, Collection Description: Portland Harbor Remedial AR File Final Remedial Investigation. 2016.
6. West JE, O'Neill SM, Ylitalo GM. Time Trends of Persistent Organic Pollutants in Benthic and Pelagic Indicator Fishes from Puget Sound, Washington, USA. Archives of environmental contamination and toxicology. 2017:1-23.
7. Lauenstein GG, Cantillo AY. Sampling and analytical methods of the NS&T program national benthic surveillance and mussel watch projects. Volume I. Overview and Summary of Methods. https://repository.library.noaa.gov/view/noaa/2893/noaa_2893_DS1.pdf. NOAA Technical Memorandum NOs ORCA. 1993;71.
