# Supplementary material for: Legacy habitat contamination as a limiting factor for Chinook salmon recovery in the Willamette Basin, Oregon, USA
Source: PLoS One. 2019 Mar 22;14(3):e0214399. doi: 10.1371/journal.pone.0214399 (PMC6430382; doi:10.1371/journal.pone.0214399)
Supplement: S2 Text — (PDF) [file pone.0214399.s005.pdf]

S2 Text. McKenzie River spring-run Chinook salmon life cycle model description.

The following is a description of the McKenzie River spring-run Chinook salmon life cycle model. It is a summary of a report with more expansive detail about the model [1].

The life cycle model is an age-structured stage-based population viability model with stochastic elements (Fig 4 in main text). It consists of an array of abundance of individuals by age (from age 1 through 6 year olds; the rows of the array) of three tributaries of the McKenzie River system that have consistent and significant fish production contributing to the population (Fig S1; South Fork McKenzie River above the reservoir at Cougar Dam, McKenzie River from below Cougar Dam to Leaburg Dam, and the McKenzie River below Leaburg Dam to the confluence with the Willamette River; the columns of the array).

Individuals in the array advanced to the next age class (row) at each annual model time step by application of survival and productivity parameters of the population which came from: studies in the basin, borrowed from similar nearby populations, derived from the model calibration process, informed from published studies of Chinook salmon, or that were inferred from expert opinion (S2 Table). The parameters consisted of: demographic rates that determined productivity, survival, and capacity; life history splits into one of several juvenile rearing strategies; ocean maturation rates; harvest. Some parameter values were unique to a particular tributary production area (e.g., egg capacities, and dam survival if fish in a tributary must navigate a project) while other parameters were shared and applied to fish from all tributary production areas (e.g., ocean maturation and survival, harvest).

Returning adults cannot reach upstream spawning areas in the South Fork McKenzie River because there is no means of adult upstream fish passage past Cougar Dam. A portion of

the adults that return for spawning in the reach below the dam are collected and either transported and released above the dam, or are used as broodstock for the hatchery supplementation program. Progeny from returning hatchery adults that did not return to the hatchery and that spawned in the wild are counted as naturally produced fish.

Reference

1. Zabel RW, Myers J, Chittaro P, Jorgensen J. Viable Salmonid Population (VSP) modeling of Willamette River spring Chinook and steelhead populations. Appendix C: Detailed biological analysis, In Willamette Valley projects configuration/operation plan (COP) Phase II report, U.S. Army Corps of Engineers, Portland District, Portland, OR.
[Main report: http://pweb.crohms.org/tmt/documents/FPOM/2010/Willamette_Coordination/Main%20Rpt%20COP%20II_Final_29Oct15.pdf]. 2015.
